# DRAIN AMYLASE ON THE FIRST POSTOPERATIVE DAY OF WHIPPLE SURGERY: WHAT VALUE IS THE BEST PREDICTOR FOR EARLY DRAIN REMOVAL?

**DOI:** 10.1590/0102-672020180001e1345

**Published:** 2018-03-01

**Authors:** Enio Campos AMICO, Ítalo Medeiros de AZEVEDO, Marcos Vinicius de Lira FERNANDES, Mariane Albuquerque REIS, Samir Assi JOÃO

**Affiliations:** 1 Department of Integrated Medicine, Federal University of Rio Grande do Norte, University Hospital Onofre Lopes, Natal, RN, Brazil

**Keywords:** Amylase, Postoperative complications, Pancreatic fistula, Pancreatoduodenectomy, Amilase, Complicações pós-operatórias, Fístula pancreática, Pancreatoduodenectomia

## Abstract

**Background:**

The value of drain amylase on the first postoperative day after pancreatic resections has been described as an efficient predictor of pancreatic fistula. In spite of this, the cut-off point below which the drains can be removed early remains controversial.

**Aim:**

Validate the use of the amylase on the 1^st^ postoperative day in the correlation with pancreatic fistula and define the value at which early drain removal is safe.

**Method:**

Were included patients undergoing Whipple surgery in the period of 2007 to 2016. Group 1 enrolled the ones who did not develop fistula and those who developed biochemical fistula for less than seven days postoperatively and group 2 included patients who developed persistent biochemical fistula between seven and 21 days and those with grade B and C fistula.

**Results:**

Sixty-one patients were included, 41 comprised group 1 and 20 group 2. The incidence of abdominal collections, need for reoperation and time of hospitalization were for group 1 and 2, respectively: 17.1%, 17.1% and 9.5 days, and 65%, 40% and 21.1 days. The median of the amylase from the drain at 1^st^ postoperative day was in group 1 and 2, respectively: 175 U/l and 3172.5 U/l (p=0.001). Using a cut-off of 180 to predict the group to which the patient would belong there was obtained sensitivity, specificity, positive predictive value and negative predictive value of 100%, 48.8%, 50% and 100% respectively.

**Conclusion:**

It was validated the cut-off value of 180 U/l as appropriate to early drain removal.

## RESUMO

**Racional:**

O valor da amilase do dreno no primeiro dia pós-operatório após ressecções pancreáticas é descrito como eficiente preditor de fístula pancreática. Entretanto, o valor abaixo do qual os drenos podem ser removidos precocemente permanece controverso.

**Objetivo:**

Validar o uso da amilase do primeiro dia pós-operatório na correlação com a fístula pancreática e definir o valor em que seja segura a retirada precoce do dreno.

**Método:**

Foram incluídos pacientes submetidos à operação de Whipple no período de 2007 a 2016. No grupo 1 entraram os que não desenvolveram fístula e os que desenvolveram fístula bioquímica por menos de sete dias de pós-operatório e no grupo 2 os que desenvolveram fístula bioquímica persistente entre 7 e 21 dias e aqueles com fístula grau B e C.

**Resultados:**

Sessenta e um pacientes foram incluídos, sendo 41 do grupo 1 e 20 do grupo 2. A incidência de coleções abdominais, necessidade de reoperação e tempo de internação foram para o grupo 1 e 2, respectivamente 17,1%, 17,1% e 9,5 dias, e 65%, 40% e 21,1 dias. A mediana da amilase no grupo 1 e 2, respectivamente foi de 175 U/l e 3172,5 U/l (p=0,001). Utilizando o ponto de corte de 180 para predizer o grupo a que o paciente pertenceria, obteve-se sensibilidade, especificidade, valor preditivo positivo e valor preditivo negativo de: 100%, 48,8%, 50% e 100% respectivamente.

**Conclusão:**

Esta amostra pôde validar o ponto de corte de 180 U/l como adequado para a retirada precoce do dreno.

## INTRODUCTION

Historically, abdominal drains have been utilized at the end of gastrointestinal surgeries with the objective of removing blood, pancreatic juices, lymph, and other secretions that could be present. Besides, it is an efficient manner to identify pancreatic fistulas (PF) and even treat them^16^. In the last 25 years, some studies have suggested that abdominal drains placed after duodenopancreatectomy can cause negative effects due to the risk of contamination of the abdominal cavity or even due to direct lesions of the intestinal loops or anastomosis^13^ ^,^ ^11^. There are few studies that have randomized the use (or not) of systematic prophylactic drains in pancreatic resections, with conflicting results^8^ ^,^ ^18^ ^,^ ^19^, which has been a limiting factor in the practice of not using drains in this type of procedure. 

Description of correlation between drain amylase in the 1^st^ postoperative day (AD1PO) after pancreatic resections and development of PF has stimulated a new approach to manage these patients. The idea is to substitute the non-utilization of drains in a systematic manner for early removal in those cases where AD1PO dosage is considered low. Several studies have attempted to define an ideal cut-off point for this scenario and variations are as wide as 5.000 U/L^6^ and 90 U/l^14^ have been suggested. 

The objective of the study presented herein is, from a cohort of patients submitted prospectively to duodenopancreatectomy, validate the use of AD1PO in the correlation with PF and define the most adequate cut-off point for early removal of drains in a group of patients.

## METHOD

The project was submitted and approved by the Human Research Ethics Committee, under nº 59716616.9.0000.5292 in the Plataforma Brasil.

Sixty-three patients were analyzed from a prospective database, who had undergone Kausch-Whipple surgery performed by the 1^st^ author of this study (ECA), for the treatment of pancreatic or peripancreatic diseases. The time period was between June 2007 and September 2016, in the following hospitals located in the city of Natal: Hospital Universitário Onofre Lopes, Liga Norte Riograndense Contra o Câncer, Casa de Saúde São Lucas and Natal Hospital Center.

The technical steps for the Whipple technique as performed by the author are detailed in previous studies^1^ ^,^ ^2^. Three techniques for pancreatojejunal anastomosis were utilized. For pancreas with main pancreatic duct ≥5 mm, two-layer pancreatojejunal terminolateral duct to mucosa anastomosis was performed. For pancreas with normal or slightly dilated (diameter <5 mm) main pancreatic duct, terminoterminal pancreatojejunal anastomosis (“telescopic”) or terminolateral pancreatojejunal anastomosis (“invagination”) were carried out, the latter performed systematically after the 33^rd^ patient from the casebook. At the end of the surgery, two drains, preferably laminar silicone drains, were placed in the cavity and exteriorized at each flank. 

In the majority of patients, subcutaneous octreotide was utilized as prophylaxis for the prevention of PF, at 0.3 mg/day, fractioned in 8 h intervals, during seven days. Debits were recorded along with amylase dosage of the drained liquid on the 1^st^, 3^rd^, 5^th^, 7^th^ and sometimes, on the 9^th^ postoperative day. The value of AD1PO vas defined from the highest amylase value of both drains, measured from a sample of liquid obtained on the first day after surgery. 

PF diagnosis utilized the 2016 revised criterion of GIEDFP^3^. PF was defined when on the 3^rd^ postoperative (PO) day the value of amylase in the drained liquid was three times higher than the normal upper limit for serum amylase. Patients that did not develop fistula or those with transient biochemical fistula (<7 days PO) were characterized as group 1, and patients with persistent biochemical fistula (between 7 and 21 days PO) and fistula grade B and C were characterized as group 2. In most cases, on the 9^th^ day after surgery, control ultrasound or tomography tests were carried out. Removal of drains occurred in those cases with low amylase values for the drained liquid (under three times the upper limit for normal serum amylase) and when image tests did not show abdominal collections. Intra-hospital mortality was defined as death occurring within 90 days of surgery.

The Receiver Operating Characteristic (ROC) curve was utilized to identify an adequate cut-off point for AD1PO and verify its predictive characteristic in patients of both groups. The ROC value was generated after sensitivity, specificity and accuracy calculations. Additionally, the cut-off point 578 U/l was also selected because of its closeness to the value utilized by Fong e cols ^9^, for comparison purposes. 

### Statistical analysis

After determination of the cut-off point, the association was verified by Fisher’s Exact test. The odds ratio (OR) was also calculated. The hypothesis tested was that AD1PO levels were different in groups 1 and 2, utilizing Mann-Whitney’s test. Chi-squared and Fisher’s tests were applied to verify the association between demographic and clinical variables with different groups. Statistical package SPSS^®^21 was utilized. A 5% significance level was applied to all tests. 

## RESULTS

From the initial sample of 63 patients, two were excluded. One had AD1PO collected incorrectly on the 2^nd^ day after surgery, and the other passed within the 1^st^ week PO. Therefore analysis covered 61 patients. Group 1 was constituted of 41 patients, of which 36 did not develop fistula and five transient biochemical fistula (grade A). Group 2 was constituted of 20 patients, of which six presented persistent biochemical fistula (grade A), eight degree B fistula, and six grade C fistula. The index of clinical fistula (grades B + C) in the overall sample was 22.9%. 

The demographic and clinical characteristics of the patients, as well as respective tests, are shown in Table 1. There was no statistical difference between groups regarding age, gender and type of illness. However, regarding the size of main pancreatic duct and type of anastomosis, a statistically significant difference was detected. All patients with dilated main pancreatic ducts as well as those submitted to duct to mucosa anastomosis belonged to group 1.

**TABLE 1:** Associations between demographic and clinical characteristics and patient groups

Variables	Group 1		Group 2		p
Age	58.0±13.06^I^		57.1±12.1^I^		0.706^II^
	n	%	n	%	
Gender					
Male	19	63.3	11	36.7	0.525^III^
Female	22	71.0	9	29.0	
Disease					
Pancreatic adenocarcinoma	17	65.4	9	34.6	0.264^III^
Papillary adenocarcinoma	12	60.0	8	40.0	
Duodenal adenocarcinoma	1	33.3	2	66.7	
Colangiocarcinoma	4	100.0	0	0.0	
Frantz	5	100.0	0	0.0	
Others	2	66.7	1	33.3	
Size of duct					
< 5	31	60.8	20	39.2	0.023^IV^
> 5	10	100.0	0	0.0	
Anastomosis*					
T-L (Invagination)	14	50.0	14	50.0	0.011^III^
T-T	17	73.9	6	26.1	
T-L (DM)	10	100.0	0	0.0	

I=mean±standard deviation; II=Mann-Whitney’s test; III= Chi-squared test; IV= Fisher’s exact test; * T-L=terminolateral; T-T=terminoterminal; T-L (DM)=terminolateral (duct to mucosa)

Regarding postoperative evolution, group 2 was associated with a higher level of general complications, postoperative collections, longer permanence of abdominal drain and hospitalization time. There was also an association between high resurgery index and patients of group 2. Nevertheless, despite all these worse result factors related to group 2, there was no statistically significant difference when comparing mortality across groups (Table 2). 

**TABLE 2:** Associations between postoperative clinical findings and patient groups, along with results for drain and hospitalization times, per group

Variable	Group 1		Group 2		p
	n	%	n	%	
Collection					
Yes	7	33.3	14	66.7	<0.001^I^
No	34	85.0	6	15.0	
Postoperative complications					
Yes	20	50.0	20.0	50.0	<0.001^II^
No	21	100.0	0	0.0	
Resurgery					
Yes	7	46.7	8	53.3	0.051^I^
No	34	73.9	12	26.1	
Time with drain (days) 9.5±2.60^III^29.1±13.58^III^ <0.001^IV^					
Hospitalization time (days) 16.7±17.9^III^34.7±16.7^III^ <0.001^IV^					
Mortality	4	9.8	1	5.0	1.000^II^

I=Chi-squared test; II=Fisher’s exact test; III= mean±standard deviation; IV=Mann-Whitney’s test

Mortality of the series was 8.19%: four patients belonged to group 1 and one patient to group 2. The mortality causes in group 1 were: kidney insufficiency due to coagulopathy; dehiscence of gastrojejunal anastomosis and sepsis, pneumonia due to broncoaspiration and necrosis of the pancreaticojejunostomy loop associated with sepsis. The only patient of group 2 passed due to necrotic pancreatitis associated with pancreatic fistula and sepsis. 

Regarding the AD1PO value, the median was 175 U/l (48.5-954) in group 1 and 2172.5 U/l (833.5-6421.0) in group 2. This difference was statistically significant (p=0.001).

The area under the ROC curve presented an exactness index of 83.0% (p=0.001). When considering a 180 U/l cut-off point for AD1PO, the following results were obtained: sensitivity 100%, specificity 48.8%, positive predictor value 50%, and negative predictor value 100%. There was an association between the arbitrated AD1PO value from the ROC curve (>180 U/l) with groups 1 and 2 (p<0.001). With a cut-off point of 578 U/l, the results were: sensitivity 80%, specificity 39%, positive predictor value 50% and negative predictor value 86.2%. There was also an association between the arbitrated AD1PO value from the ROC curve (>578 U/l) with groups 1 and 2 (p<0.003). Patients presenting AD1PO values higher than 180 U/l and 578 U/l were 2.0 and 6.25 times more likely to belong to group 2, and therefore require postoperative drainage. Table 3 shows the comparison between the two AD1PO values. 

**TABLE 3:** Precision and exactness measurements, per AD1PO cut-off point

Parameters	Cut-off point (U/l)	
	180	578
Sensitivity	100%	80%
Specificity	48.8%	39%
VPP	50%	50%
VPN	100%	86.2%

## DISCUSSION

AD1PO as a predictor of post-pancreatectomy PF was described by Yamaguchi e cols in 2003^20^. The authors analyzed the amylase values measured in abdominal drains of 26 patients submitted to pancreatectomy, and observed that values were already high on the first day PO in those who developed clinical PF after. At least three meta-analysis^10^ ^,^ ^15^ ^,^ ^21^ have validated the correlation between high AD1PO values and the development of PF. However, there is still controversy on the adequate cut-off point for AD1PO to predict PF, and on its applicability to the removal of prophylactic drains inserted after pancreatic resections. Verona’s group was the first to establish a strategy for the early removal of abdominal drains based on AD1PO values^6^. The authors carried out a prospective study on 114 patients submitted to pancreatic resection and randomized those with AD1PO <5000 U/l to remove the drain on the 3^rd^ or 5^th^ day PO. Early removal of drains was associated with a lower rate of pancreatic fistula (1.8% vs. 26%), lower rate of abdominal complications (12.2% vs. 52.6%), lower rate of pulmonary complications (26.3% vs. 52.6%), shorter hospitalization times (8.7 (±4) vs. 10.8 (±6.9) and lower hospital readmission rates (0% vs. 8.8%). Although the study suggested that early removal of abdominal drains was safe with AD1PO under 5000 U/l, other authors reported hypothetical PF indexes between 25-48% if drains were removed according to this suggestion^7^ ^,^ ^12^.

For those patients submitted to duodenopancreatectomy and that received prophylactic drains, the most important question is to know which patients will not develop PF - and not the other way around. On one hand, knowing which patients will not develop PF is useful because it allows for early drain removal, which leads to shorter hospitalization times. On the other hand, predicting which patients will develop PF after surgery, when the abdominal cavity is being drained, is not as relevant because PF can be easily diagnosed by measuring amylase in the drained liquid. In most cases, treatment is simple maintenance of drains, associated with fasting and nutritional support. For this reason the study herein presented analyzed two low AD1PO cut-off points (180 and 578) that were associated with higher specificity and consequently, with a high negative predictive factor, in detriment to a high cut-off point associated with higher sensitivity to PF diagnosis. Another reason for the selection is related to the impossibility of obtaining daily interventionist radiology services in the public hospital where the majority of the patients studied underwent surgery. These services include percutaneous drainage of abdominal collections/abscesses, which is indicated in urgent cases where there are abdominal complications as a consequence of undrained anastomotic leaks. 

The methodology of this study utilized the PF definition based on the revised classification by ISGPS^5^, which no longer considers grade A of the previous classification to be a true PF^4^. However, as this study aimed at identifying a group of patients in which early removal of drains could be beneficial, it was important to include not only the concept of PF, but also grades B and C and those patients with biochemical fistula that persisted with amylase-rich debit between 7-21 days PO. In these patients, early removal of drains would increase susceptibility to abdominal collections or abscesses. With the 180 U/l cut-off point, an excellent correlation was obtained between AD1PO and absence of fistula. Therefore it was possible to identify 1/3 of patients that would have been benefitted by early drain removal. Besides, with the same cut-off, none of the patients that developed clinically relevant fistula or even persistent biochemical fistula would have had their drains removed early. 

Data obtained herein were capable of validating previous publications that utilized low cut-off points^7^ ^,^ ^12^ ^,^ ^14^ ^,^ ^17^. Differently from these studies, however, the first series was entirely prospective herein, including only patients submitted consecutively to duodenopancreatectomy by a single team of surgeons, where PF definition followed ISGPS criteria. The cut-off point 180 U/l obtained herein was lower than the one found by Fong et al^9^ at the Massachusetts General Hospital. They divided the study in two cohorts: the first involved 126 patients, and the 612 U/l cut-off point was defined as the one presenting best accuracy, sensitivity and specificity; the second had the objective of validating a 600 U/l cut-off point, involving 369 patients submitted to duodenopancreatectomy within January 2009 and December 2012. Almost two-thirds of patients (62.1%) presented AD1PO lower than 600 U/l and only two PF cases (0.9%) were diagnosed. This was different than the other patients with AD1PO of at least 600 U/l, with PF incidence of 31.4%. When considering herein a cut-off point close to Fong et al., 578 U/l, 13.8% of patients with PF diagnosis would have been affected by early removal of drains (negative predictor value: 86.2%). In the reality considered herein, there is no non-invasive treatment for intracavitary collections/abscesses available all the time at the public hospital, and therefore increasing the cut-off point would be a high price to pay. 

This study presents some limitations. The first concerns the low number of included patients, which did not hinder statistical analysis. The second is related to the non-verification of the real benefits of drains in patients who developed PF, as all patients received drains. The high index of abdominal collections (65%) and necessity of re-surgery (40%) in group 2 suggested that, for some patients, drains were not totally efficient; nevertheless, the objective of the study was to find a group of patients in which drains could be removed early and not confirm the non-efficiency of drains in the group that developed PF.

## CONCLUSION

Was validated the 180 U/l cut-off point as adequate to define those patients in which abdominal drains can be removed early after duodenopancreatectomy. At the same time it is recognized that there is no ideal cut-off point to be utilized uniformly in all services. The ideal cut-off point must vary in accordance with the specifications of the service and compensate for the risk of undrained PF with the benefits of a faster recovery in a higher number of patients. 
